# Negative associations between macronutrient quality index and lung cancer incidence and mortality: results from the prostate, lung, colorectal, and ovarian cancer screening trial

**DOI:** 10.3389/fnut.2025.1647438

**Published:** 2025-09-22

**Authors:** Dazhan Feng, Heng Su, Qi Wei, Yi Xiao, Haitao Gu, Linglong Peng, Yuxiang Luo, Ling Xiang, Junxia Xue, Yunhao Tang, Citong Zhang, Dengliang Liu

**Affiliations:** ^1^Department of Gastrointestinal Surgery, The Second Affiliated Hospital of Chongqing Medical University, Chongqing, China; ^2^Department of General Surgery, the Renmin Hospital of Wushan County, Chongqing, China; ^3^Erasmus University Medical Center, Rotterdam, Netherlands; ^4^Department of Clinical Nutrition, The Second Affiliated Hospital of Chongqing Medical University, Chongqing, China; ^5^Department of Radiation Oncology, Senior Department of Oncology, the Fifth Medical Center of PLA General Hospital, Beijing, China; ^6^Department of Oral Comprehensive Therapy, Hospital of Stomatology, Jilin University, Changchun, China; ^7^Department of Gastrointestinal Surgery, Chongqing Jiulongpo People's Hospital, Chongqing, China; ^8^Department of General Surgery, Xipeng Town Health Center of Jiulongpo District, Chongqing, China

**Keywords:** macronutrient quality index, cancer prevention, epidemiology, lung cancer, cohort study

## Abstract

**Background:**

Prior research has not examined the connection between the quality of macronutrients and the occurrence as well as fatality rates of lung cancer (LC). Consequently, to delve deeper into the correlations between macronutrient quality and the likelihood of developing LC, we carried out an extensive, long-term prospective cohort study of 101,755 American adults from the Prostate, Lung, Colorectal, and Ovarian (PLCO) Cancer Screening Trial.

**Methods:**

Our research cohort comprised 154,887 adults, aged between 55 and 74, who were enrolled from 10 screening facilities across the United States. The macronutrient quality index (MQI) was derived from participants’ responses to a dietary history questionnaire (DHQ). To quantify the strength and precision of the relationships between MQI and the incidence as well as mortality of LC, we employed Cox proportional hazards regression modeling to estimate hazard ratios (HRs) alongside their corresponding 95% confidence intervals (CIs). Additionally, we conducted subgroup analyses to scrutinize whether the observed link between MQI and LC risk was subject to modification by potential confounding variables. To reinforce the reliability of our results, sensitivity analyses were also carried out.

**Results:**

Over an average follow-up period spanning 8.82 ± 1.95 years (accumulating to 897,809 person-years of observation), we recorded 1,706 LC diagnoses, encompassing 1,464 cases of non-small cell lung cancer (NSCLC) and 242 cases of small cell lung cancer (SCLC). Additionally, there were 1,217 deaths attributed to LC, with 1,005 NSCLC-related and 212 SCLC-related fatalities. Our results demonstrate a distinct, statistically significant inverse association between a higher MQI and both a reduced incidence (HR Q4 vs. Q1: 0.65; 95% CI: 0.56–0.76; *p* < 0.001 for trend) and decreased mortality (HR Q4 vs. Q1: 0.71; 95% CI: 0.60–0.84; *p* < 0.001 for trend) of LC. This inverse relationship held true for both NSCLC and SCLC subtypes. The robustness of the associations between MQI and the incidence as well as mortality of LC was solidly affirmed through sensitivity analyses.

**Conclusion:**

Our research outcomes imply that prioritizing the intake of higher-quality macronutrients could serve as a viable strategy to mitigate LC risk within the American population.

## 1 Introduction

Lung cancer (LC) remains the leading cause of cancer-related deaths worldwide, accounting for approximately 12.4% of all cancer diagnoses and causing over 1.8 million deaths globally in 2022 (1). In the US, projections estimate around 226,650 new cases and 124,730 deaths by 2025, with those aged 50 and older being disproportionately affected (2). While developing countries exhibit a notably higher prevalence of LC, attributed to elevated smoking rates and lax tobacco control, its etiology extends beyond well-recognized risk factors such as tobacco use, radon exposure, air pollution, occupational hazards, and genetic predisposition (3–5). Given the complex mechanisms involved in LC development, further investigation into additional potential risk factors is imperative to enhance preventive strategies.

Recent scientific advancements have underscored the crucial impact of dietary patterns as modifiable lifestyle elements on the development of LC. Empirical data indicate that suboptimal dietary habits could be responsible for more than a third of LC cases and related fatalities (6, 7). The quality and origin of macronutrients, encompassing proteins, carbohydrates, and fats, serve as fundamental components in assessing dietary quality, with their links to LC risk being intricate and multifaceted. Poor-quality proteins derived from animal sources, typically rich in saturated fats, cholesterol, and carcinogenic heterocyclic amines, may stimulate uncontrolled cell growth, thereby promoting lung tumor formation (8, 9). Conversely, high-quality plant-sourced proteins, packed with dietary fiber, antioxidants, and beneficial phytochemicals, could potentially offer protective benefits against LC by mitigating inflammation and oxidative stress (10, 11). The quality of carbohydrates and fats is equally paramount in this context. Carbohydrates with a high glycemic index can disrupt insulin homeostasis, potentially creating an environment conducive to tumor progression (12, 13). On the other hand, specific fatty acid profiles, such as those abundant in *ω*-3 polyunsaturated fatty acids, have been correlated with a lower risk of LC (14, 15). However, conventional research approaches that primarily concentrate on isolated nutrients or overall intake levels have neglected the synergistic effects and quality disparities among macronutrients, leading to inconsistent and often conflicting research outcomes (16, 17).

To bridge critical research gaps regarding the intricate relationship between dietary factors and LC, the macronutrient quality index (MQI), an all – encompassing and multi-dimensional analytical tool, was developed (18, 19). It synthesizes nutrient ratios, fatty acid profiles, and food-source quality to holistically evaluate dietary patterns. While existing indices like food-group-based scoring systems provide limited insights, they fail to adequately capture the subtleties of macronutrient quality, especially lacking LC-specific, outcome-oriented assessment methods (20, 21). Building on this, a large-scale, prospective investigation was initiated, with the primary aim of elucidating the connection between macronutrient quality and LC risk. The MQI was rigorously employed to assess macronutrient quality in a study population specifically comprising Americans aged 55–74 years. Considering the well-established heterogeneity of LC, an in-depth analysis of its two major histological subtypes, NSCLC and SCLC, was conducted to ascertain whether the associations between macronutrient quality and LC risk differed across these distinct histological categories. Given the significant public health burden of LC in the United States, which profoundly affects individual health outcomes and the broader healthcare economy, the findings of this study hold substantial promise for informing the development of evidence-based preventive strategies to reduce the incidence and burden of this life-threatening disease.

## 2 Method

### 2.1 Study design

This analysis focuses on participants from the Prostate, Lung, Colorectal, and Ovarian (PLCO) Cancer Screening Trial, a large-scale randomized clinical trial initiated and funded by the National Cancer Institute (NCI) between 1993 and 2001 (22). The inclusion criteria for participant recruitment were as follows: (1) age 55–74 years at enrollment; (2) no prior history of prostate, lung, colorectal, or ovarian cancer; and (3) voluntary provision of informed consent after thorough explanation of study objectives, procedures, and potential risks/benefits. Based on these criteria, a total of 154,887 men and women were recruited across 10 screening centers nationwide (22). Upon enrollment, participants were randomly allocated to one of two arms: the standard medical care group (control arm) or the group receiving enhanced cancer screening protocols (intervention arm). The trial’s protocol was subject to a stringent ethical review process and obtained approval from the institutional review boards at both the NCI headquarters and the participating study sites. A comprehensive account of the PLCO trial’s methodological design, encompassing statistical power calculations and participant recruitment strategies, has been documented in previous publications.

### 2.2 Data collection and covariates assessment

In the PLCO cancer screening trial, demographic data were collected through a self-administered baseline questionnaire (BQ). This study encompassed diverse baseline characteristics, including age, gender, ethnicity, marital status, educational level, trial group assignment (control or intervention arm), family cancer history, and past medical conditions like hypertension, chronic bronchitis, emphysema, and diabetes. Anthropometric data focused on body mass index (BMI), calculated as weight (kg) divided by height squared (m^2^), and weight change, defined as the difference between baseline weight and self-reported weight at age 20. Two key parameters were used to assess smoking status: smoking history (categorized as never-smoker, former smoker, or current smoker) and smoking intensity (classified as 0, 1–20, or >20 cigarettes per day). Additionally, data on alcohol consumption history were gathered. Dietary information was obtained via the Diet History Questionnaire (DHQ), a comprehensive self-administered food frequency questionnaire (FFQ) administered post-enrollment in the PLCO trial. The 137-item DHQ systematically evaluated dietary intake over the past year, covering the frequency of consuming various food groups (e.g., meats, vegetables, fruits), dietary supplement use, and portion sizes (23).

### 2.3 Population for analysis

**Figure 1:**
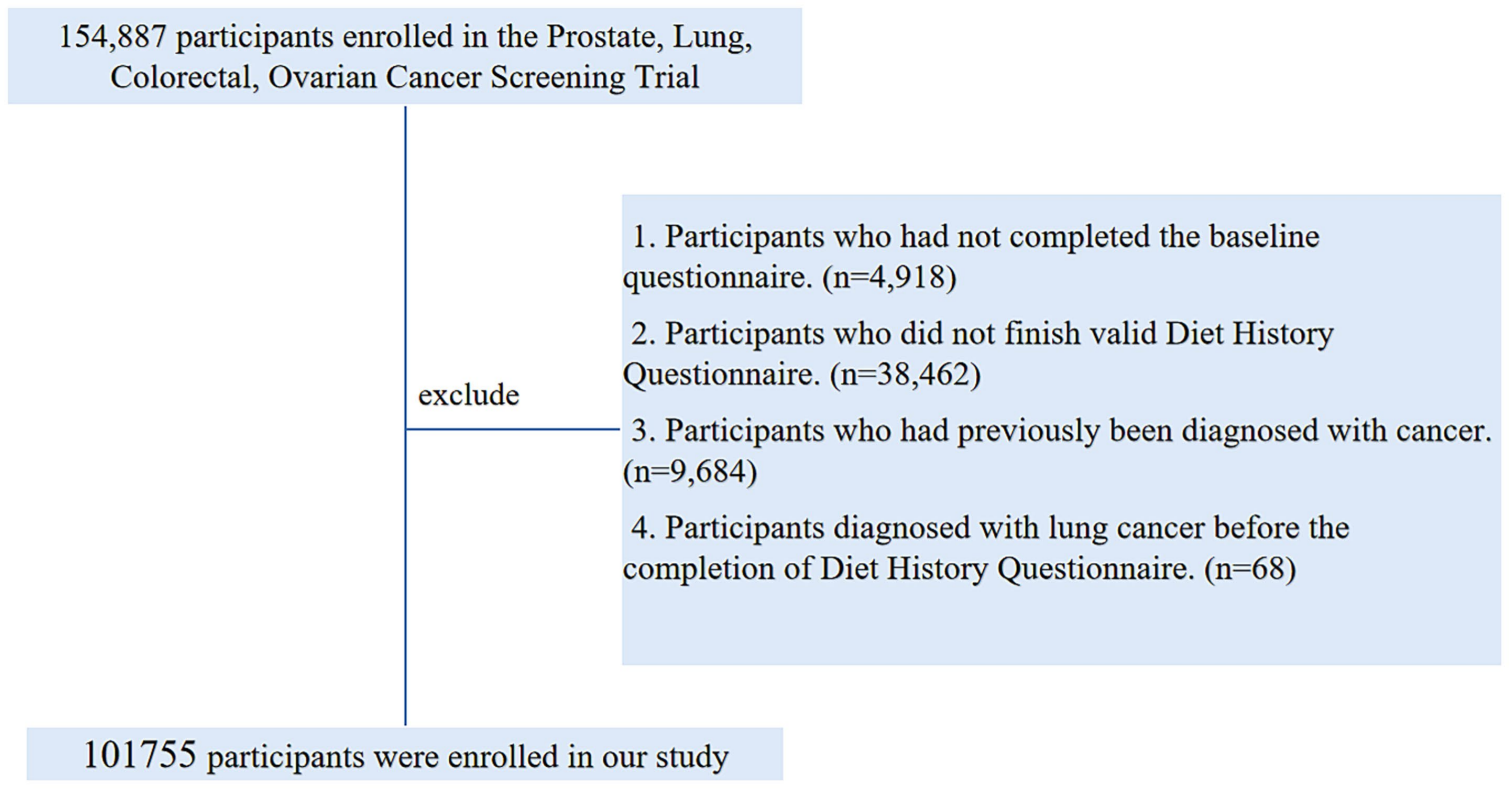
The flow chart of identifying eligible subjects. PLCO, prostate, lung, colorectal, and ovarian; BQ, baseline questionnaire; DHQ, diet history questionnaire.

At the study’s baseline, we established a set of exclusion criteria to define the final sample for analysis: (1) Participants who had not completed the baseline questionnaire (*n* = 4,918); (2) Participants who did not finished valid Diet History Questionnaire [those failure to return DHQ responses, those lacking a completion date, those completed after the death date, those with a high frequency of missing responses (≥8), or those with extremely high energy intake values (the first or last percentile)] (*n* = 38,462); (3) Participants who had previously been diagnosed with cancer (*n* = 9,684); (4) Participants diagnosed with LC before the completion of Diet History Questionnaire (n = 68). Ultimately, our analytic sample consisted of 101,755 individuals (49,496 males and 52,259 females) (Figure 1).

### 2.4 Calculation of MQI

The MQI combines three different sub-indices to assess macronutrient intake quality. They are the Carbohydrate Quality Index (CQI), Fat Quality Index (FQI), and Healthy Plate Protein Quality Index (HPPQI).

The CQI, based on participant quintiles, consists of four 1–5 scored components. Glycemic index (GI) is reverse-scored to prioritize low-GI diets that lower metabolic risk. Total dietary fiber intake is directly scored for its satiety-promoting and gut-health benefits. The solid/liquid carbohydrate ratio distinguishes whole foods from processed drinks, favoring solid sources. The whole grain/total grain ratio promotes whole grains over refined ones, in line with chronic disease prevention evidence (24–28). Summing these gives a CQI of 4–20.

The FQI functions as a fat quality indicator, assigning lower scores to diets rich in pro-inflammatory fats and higher scores to those abundant in cardioprotective unsaturated fats. This guarantees a balanced weighting across fatty acid categories for a fair assessment of lipid intake (25, 27). It’s calculated via the ratio:

*FQI = (Monounsaturated + Polyunsaturated)/(Saturated + Trans Fatty Acids) (MUFA + PUFA)/(SFA + TFA)*.

The HPPQI evaluates protein source quality through a ratio that prioritizes seafood, poultry, legumes, and nuts—rich in high-quality protein, essential amino acids, and bioactives—over red/processed meats and cheese (29, 30). It conforms to global dietary guidelines advocating plant-based protein options. The HPPQI is calculated with the ratio (31):

*HPPQI = (seafood + poultry + pulses + nuts)/(red and processed meats + cheese)*.

Each sub-index equally contributes to the MQI total. Participants get quintile-based scores (1–5) for each. The MQI is the sum of these, with a 3–15 range (32). This enables detailed dietary quality assessment across macronutrients, offering insights into diet-health links. Validated in large cohorts, it’s useful for evaluating dietary patterns’ ties to chronic diseases (32, 33).

### 2.5 Ascertainment of outcome events

In the PLCO trial, LC case ascertainment hinged on the annual update process. Participants were mandated to submit comprehensive cancer data, like diagnosis specifics, date, treating facility, and physician contact info. Reported cases were validated via medical record scrutiny. Their survival status was tracked through annual updates; for non-responders, the team conducted follow-ups by phone and email. For case validation, the study used ICD-O-2 for cancer diagnosis and ICD-9-coded death certificates for cause-of-death determination.

### 2.6 Statistical analysis

The study encountered missing data across multiple variables. For categorical covariates with missing rates below 5%, such as race, marital status, education level, aspirin dose, diabetes history, emphysema/chronic bronchitis/hypertension history, X-ray exposure history, family LC history, smoking status, and alcohol consumption history, the mode was used for imputation. Continuous covariates with missing rates under 5%, namely BMI and smoking pack-years, were imputed with the median (34). Detailed imputation details and missing proportions for each variable are in Supplementary Table 1.

**Figure 2:**
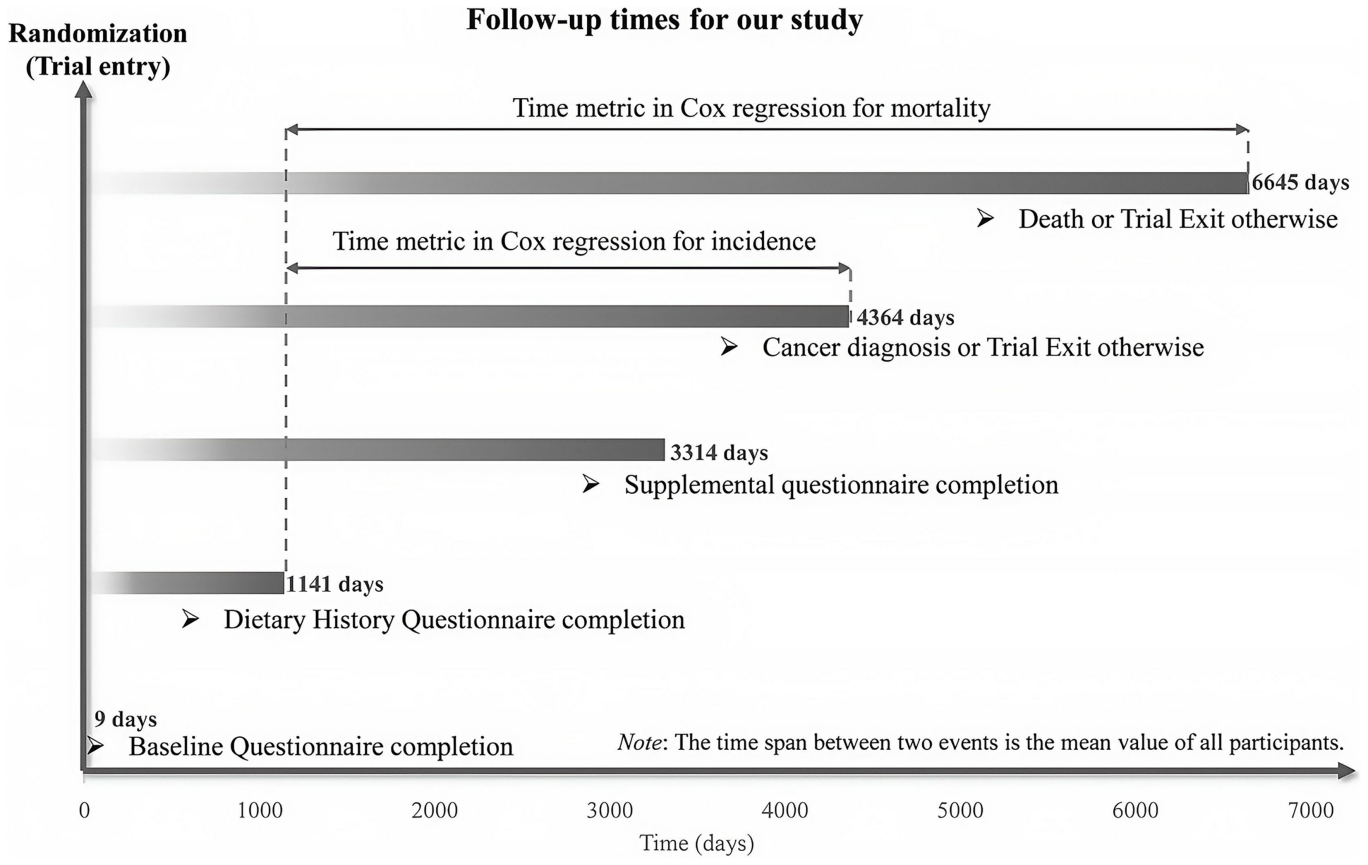
The timeline and follow-up scheme of our study.

In this research, the time from completing the DHQ to a LC-related event (diagnosis or death) was recorded in days. For primary outcomes, the follow-up span was from DHQ completion to the earliest of LC diagnosis, death, loss of contact, or December 31, 2009 (the cut-off for cancer incidence tracking). Secondary outcome follow-up ended in 2018, as stated on the PLCO website^1^ (Figure 2). Cox proportional hazards models were established to estimate the risk ratios (HRs) and 95% confidence bounds (CIs) for the link between MQI and the outcomes, using the follow-up duration as the time variable. MQI was categorized into quartiles, with the lowest quartile as the reference. To probe linear trends, continuous variables were formed from the median MQI within each quartile; the *P*-value indicated the trend’s significance. Potential confounders were picked based on known LC risk factors or the researchers’ clinical insights (35). These were added to the Cox models to reduce confounding bias. Model 1 adjusted for basic demographics (sex, age, race, education, and marital status). Model 2 further accounted for lifestyle and clinical factors (BMI, smoking, daily cigarette count, alcohol history, hypertension, emphysema/chronic bronchitis history, aspirin use, diabetes, and family LC history) along with the trial group. Restricted cubic spline (RCS) models were used to depict the MQI-LC incidence and mortality relationships, using the median MQI as the reference in each case. Non-linearity was assessed by testing if the second spline term’s coefficient was zero (36, 37). The same analyses were also carried out for NSCLC and SCLC. In addition, we employed Kaplan–Meier survival curves to describe the association between the MQI and the incidence/mortality rates of LC. Moreover, we conducted a log-rank test to compare the differences in survival curves among different MQI level groups.

We conducted prespecified subgroup analyses to examine whether key factors might modify the associations between the MQI and LC incidence and mortality. Subgroups were classified based on: demographic traits (age >65 vs. ≤65 yrs.; male vs. female; White vs. non-White race; education: below college, college graduate, postgraduate; married vs. unmarried), health status (diabetes: yes/no; hypertension: yes/no; baseline BMI ≤ 30 vs. >30 kg/m^2^), family and medical background (family LC history: absent/present; emphysema history: yes/no; chronic bronchitis history: yes/no), and lifestyle habits (smoking: never vs. current/former; daily cigarettes: 0, 1–20, >20; aspirin use: no/yes; alcohol consumption history: yes/no). To detect potential false subgroup effects, we assessed interaction *P*-values by contrasting models with and without interaction terms.

To augment the credibility of our findings, we conducted a series of sensitivity analyses (35, 38, 39):

1. To tackle potential reverse causality, we did sensitivity analyses by excluding cases in the first 2 and 4 years of follow-up.
2. Excluded individuals with extreme energy intake (energy intake >4,000 kcal/day or <500 kcal/day).
3. Excluded individuals with extreme BMI values (the lowest 1% and the highest 1%).
4. To augment the statistical robustness of the study, smoking pack-years were incorporated as an adjusted variable in lieu of daily cigarette consumption.
5. Since certain medical conditions raise the risk of LC, we excluded those with diabetes mellitus and respiratory comorbidities (emphysema and chronic bronchitis).

All statistical analyses were carried out using R software version 4.3.1, with two-tailed *p* < 0.05 as the level of statistical significance.

## 3 Results

### 3.1 Participant baseline features

In this research, participants were stratified into four quartiles according to their MQI: Quartile 1 (3–7), Quartile 2 (8–9), Quartile 3 (10–11), and Quartile 4 (12–15). As indicated in Table 1, individuals in the highest quartile (Q4) exhibited distinct demographic and health-related characteristics. Specifically, they were more inclined to be female, non-white, and possess a higher level of education. Conversely, they were less likely to be married, use aspirin, or have been diagnosed with conditions such as diabetes, hypertension, chronic bronchitis, or emphysema. Additionally, participants in Q4 tended to have a lower smoking intensity and presented with a lower and less fluctuating BMI.

**Table 1:** Baseline characteristics of study population according to MQI.

Characteristics	Overall	Quartiles of overall MQI			
		Quartile 1	Quartile 2	Quartile 3	Quartile 4
Number of participants	10,1755	36,216	23,771	21,089	20,679
Age	62.40 ± 5.28	62.10 ± 5.18	62.36 ± 5.24	62.51 ± 5.29	62.65 ± 5.41
Sex					
Male	49,496 (48.64%)	21,300 (58.81%)	11,385 (47.89%)	8,966 (42.52%)	7,845 (37.94%)
Female	52,259 (51.36%)	14,916 (41.19%)	12,386 (52.11%)	12,123 (57.48%)	12,834 (62.06%)
Race					
White	94,066 (92.44%)	34,481 (95.21%)	22,062 (92.81%)	19,077 (90.46%)	18,446 (89.20%)
Non-white	7,689 (7.56%)	1735 (4.79%)	1709 (7.19%)	2012 (9.54%)	2,233 (10.80%)
Education level					
College below	64,953 (63.83%)	25,281 (69.81%)	15,370 (64.66%)	12,777 (60.59%)	11,525 (55.73%)
College graduate	17,848 (17.54%)	5,670 (15.66%)	4,174 (17.56%)	3,955 (18.75%)	4,049 (19.58%)
Postgraduate	18,954 (18.63%)	5,265 (14.54%)	4,227 (17.78%)	4,357 (20.66%)	5,105 (24.69%)
Marriage					
Married	79,826 (78.45%)	28,658 (79.13%)	18,933 (79.65%)	16,532 (78.39%)	15,703 (75.94%)
Unmarried	21,929 (21.55%)	7,558 (20.87%)	4,838 (20.35%)	4,557 (21.61%)	4,976 (24.06%)
Diabetes history					
No	94,949 (93.31%)	33,670 (92.97%)	22,166 (93.25%)	19,673 (93.29%)	19,440 (94.01%)
Yes	6,806 (6.69%)	2,546 (7.03%)	1,605 (6.75%)	1,416 (6.71%)	1,239 (5.99%)
Aspirin use history					
No	53,953 (53.02%)	19,163 (52.91%)	12,571 (52.88%)	11,148 (52.86%)	11,071 (53.54%)
Yes	47,802 (46.98%)	17,053 (47.09%)	11,200 (47.12%)	9,941 (47.14%)	9,608 (46.46%)
X-ray history					
No	46,303 (45.51%)	16,518 (45.61%)	10,727 (45.12%)	9,630 (45.66%)	9,428 (45.58%)
Once	32,918 (32.35%)	11,728 (32.38%)	7,692 (32.35%)	6,758 (32.03%)	6,740 (32.60%)
More than once	18,377(18.06%)	6,519 (17.99%)	4,367 (18.37%)	3,802 (18.02%)	3,689 (17.84%)
Possibly	4,157(4.08%)	1,451 (4.02%)	985 (4.16%)	899 (4.29%)	822 (3.98%)
Family history of lung cancer					
No	88,738 (87.21%)	31,352 (86.57%)	20,747 (87.28%)	18,433 (87.41%)	18,206 (88.04%)
Yes	10,569 (10.39%)	3,856 (10.65%)	2,453 (10.32%)	2,202 (10.44%)	2058 (9.95%)
Possibly	2,448 (2.41%)	1,008 (2.78%)	571 (2.40%)	454 (2.15%)	415 (2.01%)
Chronic bronchitis history					
No	97,423 (95.74%)	34,548 (95.39%)	22,786 (95.86%)	20,214 (95.85%)	19,875 (96.11%)
Yes	4,332 (4.26%)	1,668 (4.61%)	985 (4.14%)	875 (4.15%)	804 (3.89%)
Emphysema history					
No	99,611 (97.89%)	35,188 (97.16%)	23,281 (97.94%)	20,750 (98.39%)	20,392 (98.61%)
Yes	2,144 (2.11%)	1,028 (2.84%)	490 (2.06%)	339 (1.61%)	287 (1.39%)
Hypertension history					
No	68,707 (67.52%)	24,058 (66.43%)	15,849 (66.67%)	14,307 (67.84%)	14,493 (70.09%)
Yes	33,048 (32.48%)	12,158 (33.57%)	7,922 (33.33%)	6,782 (32.16%)	6,186 (29.91%)
Family history of cancer					
No	44,899 (44.12%)	16,193 (44.71%)	10,497 (44.16%)	9,206 (43.65%)	9,003 (43.54%)
Yes	56,856 (55.88%)	20,023 (55.29%)	13,274 (55.84%)	11,883 (56.35%)	11,676 (56.46%)
Arm					
Intervention	51,817 (50.92%)	18,244 (50.38%)	12,023 (50.58%)	10,671 (50.60%)	10,879 (52.61%)
Control	49,938 (49.08%)	17,972 (49.62%)	11,748 (49.42%)	10,418 (49.40%)	9,800 (47.39%)
Smoking status					
No	48,580 (47.74%)	15,522 (42.86%)	11,413 (48.01%)	10,577 (50.15%)	11,068 (53.52%)
Current/former	53,175 (52.26%)	20,694 (57.14%)	12,358 (51.99%)	10,512 (49.85%)	9,611 (46.48%)
Body mass index at baseline (kg/m^2^)	27.22 ± 4.79	27.93 ± 4.83	27.39 ± 4.80	26.90 ± 4.71	26.13 ± 4.54
Weight fluctuation^a^	2.84 ± 0.82	2.96 ± 0.82	2.87 ± 0.82	2.79 ± 0.82	2.66 ± 0.80
Smoking pack-years	17.65 ± 26.59	21.86 ± 29.73	17.68 ± 26.49	15.30 ± 24.25	12.66 ± 21.53
Daily cigarette consumption					
0	48,685 (47.85%)	15,571 (42.99%)	11,434 (48.10%)	10,595 (50.24%)	11,085 (53.61%)
1–20	33,218 (32.65%)	11,891 (32.83%)	7,715 (32.46%)	6,966 (33.03%)	6,646 (32.14%)
>20	19,852 (19.51%)	8,754 (24.17%)	4,622 (19.44%)	3,528 (16.73%)	2,948 (14.26%)
History of alcohol consumption					
No	27,757 (27.28%)	9,460 (26.12%)	6,302 (26.51%)	5,836 (27.67%)	6,159 (29.78%)
Yes	73,998 (72.72%)	26,756 (73.88%)	17,469 (73.49%)	15,253 (72.33%)	14,520 (70.22%)

Descriptive statistics are presented as (mean ± standard deviation) and number (percentage) for continuous and categorical.

^a^Weight fluctuation was defined as the participant's baseline weight minus weight at age 20.

### 3.2 Association between LC incidence and MQI

**Figure 3:**
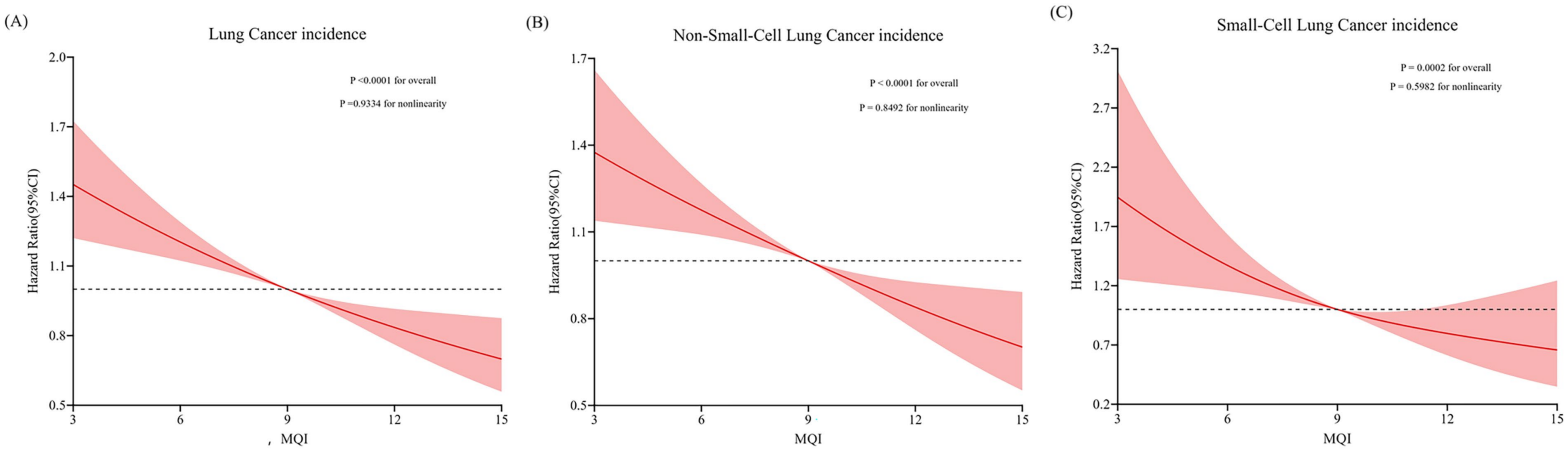
Restricted cubic spline (RCS) model on the association of MQI with incidence of LC **(A)**, NSCLC **(B)** and SCLC **(C)**. Hazard ratio was adjusted for age (years), sex (male, female), race (white and non-white), education levels (college below, college graduate, postgraduate), marital status (married, unmarried), smoking status (never, currently/ever), number of cigarettes smoked (0, 1–20, >20 cigarettes/day), history of alcohol consumption (yes, no), history of emphysema (yes, no), chronic bronchitis history (yes, no), body mass index (kg/m^2^), trial arm (intervention, control), aspirin use (yes, no), history of diabetes (yes, no), history of hypertension (yes, no) and family history of lung cancer (yes, no).

During an average 8.82 ± 1.95-year follow-up (897,809 person-years), 1,706 LC cases were identified, including 1,464 NSCLC and 242 SCLC cases, with an overall incidence rate of about 19.00 per 10,000 person-years. As shown in Table 2, after adjusting for potential confounders, Cox regression analysis revealed a significant inverse link between higher MQI and LC incidence (HR Q4 vs. Q1: 0.65; 95% CI: 0.56, 0.76. *p* < 0.001 for trend). Similar negative correlations were observed in the association between MQI and the incidence of NSCLC (HR Q4 vs. Q1: 0.66; 95% CI: 0.56, 0.78; *p* < 0.001 for trend) and SCLC incidence (HR Q4 vs. Q1: 0.61; 95% CI: 0.42, 0.90; *p* = 0.002 for trend). The RCS model revealed linear relationships between MQI and the incidence of overall LC and both NSCLC and SCLC (Figure 3). The survival curve results indicate that there are significant differences in survival curves among different MQI level groups (*p* < 0.0001). As time progresses, the high-MQI group demonstrates a generally lower incidence of lung cancer overall (Supplementary Figure 1).

**Table 2:** Hazard ratios of the association between MQI and lung cancer incidence.

Quartiles of MQI	Cases	Person-years	Incidence rate per 10,000 person-years (95% confidence interval)	Hazard ratio (95% confidence interval) by MQI		
				Unadjusted	Model 1^a^	Model 2^b^
Lung cancer						
Quartile 1	767	315946.0	24.28 (22.62,26.05)	1.000 (reference)	1.000 (reference)	1.000 (reference)
Quartile 2	406	210154.2	19.32 (17.53,21.29)	0.79 (0.70,0.90)	0.84 (0.74,0.95)	0.88 (0.78,0.99)
Quartile 3	294	187748.6	15.66 (13.97,17.55)	0.64 (0.56,0.74)	0.70 (0.61,0.80)	0.75 (0.66,0.86)
Quartile 4	239	183960.7	12.99 (11.45,14.75)	0.53 (0.46,0.62)	0.59 (0.51,0.69)	0.65 (0.56,0.76)
*p* for trend				<0.001	<0.001	<0.001
Non-small-cell lung cancer						
Quartile 1	639	315946.0	20.23 (18.72, 21.85)	1.000 (reference)	1.000 (reference)	1.000 (reference)
Quartile 2	359	210154.2	17.08 (15.41, 18.94)	0.84 (0.74,0.96)	0.89 (0.78,1.01)	0.93 (0.82,1.06)
Quartile 3	261	187748.6	13.90 (12.32, 15.69)	0.69 (0.59,0.79)	0.74 (0.64,0.86)	0.80 (0.69,0.92)
Quartile 4	205	183960.7	11.14 (9.72, 12.78)	0.55 (0.47,0.64)	0.61 (0.52,0.71)	0.66 (0.56,0.78)
*p* for trend				<0.001	<0.001	<0.001
Small-cell lung cancer						
Quartile 1	128	315946.0	4.05 (3.41, 4.82)	1.000 (reference)	1.000 (reference)	1.000 (reference)
Quartile 2	47	210154.2	2.24 (1.68, 2.97)	0.55 (0.39,0.77)	0.59 (0.42,0.83)	0.63 (0.45,0.88)
Quartile 3	33	187748.6	1.76 (1.25, 2.47)	0.43 (0.30,0.63)	0.49 (0.33,0.72)	0.54 (0.36,0.79)
Quartile 4	34	183960.7	1.85 (1.32, 2.58)	0.45 (0.31,0.66)	0.54 (0.37,0.79)	0.61 (0.42,0.90)
*p* for trend				<0.001	<0.001	0.002

^a^Model 1 was adjusted with age (continuous), sex (male, female), race (white, non-white), education levels (college below, college graduate, postgraduate) and marriage (married, unmarried).

^b^Model 2 was adjusted for model 1 plus BMI at baseline (continuous), trial arm (intervention, control), smoking status (never, current or former), daily cigarette consumption (0, 1–20, >20), history of alcohol consumption (yes, no), aspirin use (no, yes), family history of lung cancer (no, yes, possibly), history of hypertension (no, yes), history of diabetes (no, yes), history of chronic bronchitis (no, yes) and history of emphysema (no, yes).

Subgroup analysis detected a significant interaction (*p* < 0.05) regarding the influence of smoking status on the inverse relationship between MQI and LC incidence. This implies that a higher MQI confers a more pronounced protective effect in mitigating LC incidence among current or former smokers (HR Q4 vs. Q1: 0.62; 95% CI: 0.53, 0.73; *p* < 0.001 for trend) compared to individuals with no smoking history (HR Q4 vs. Q1: 1.13; 95% CI: 0.70, 1.82; *p* = 0.899 for trend). In other subgroup analyses, no substantial differences in the inverse relationship between MQI and LC incidence were found (all interaction *p*-values > 0.05), as detailed in Supplementary Table 2. Moreover, sensitivity analyses consistently validated the association between MQI and LC incidence, as presented in Table 3.

**Table 3:** The sensitivity analyses between MQI and lung cancer incidence.

Categories	HR^e^ (Quartile 4 vs. Quartile 1, 95% CI)	*p* for trend
Exclude extreme energy intake^a^	0.66 (0.56,0.77)	<0.001
Exclude extreme BMI^b^	0.65 (0.56,0.76)	<0.001
Excluding Patients diagnosed within 2 years	0.64 (0.54,0.75)	<0.001
Excluding Patients diagnosed within 4 years	0.59 (0.49,0.71)	<0.001
Excluding patients with diabetes or respiratory comorbidities^c^	0.62 (0.53,0.74)	<0.001
Replace the Indicator of cigarettes smoked^d^	0.73 (0.63,0.85)	<0.001

^a^Extreme energy intake was defined as energy intake >4,000 kcal/day or <500 kcal/day.

^b^BMI defined as body mass index at baseline (kg/m^2^).

^c^Respiratory comorbidities included contain emphysema and chronic bronchitis.

^d^Adjusting for pack-years of smoking (continuous) instead of daily cigarette consumption (0, 1–20, or >20).

^e^HR was adjusted for age (years), sex (male, female), race (white and non-white), education levels (college below, college graduate, postgraduate), marital status (married, unmarried), BMI at baseline (continuous), trial arm (intervention, control), smoking status (never, current or former), daily cigarette consumption (0, 1–20, >20), history of alcohol consumption (yes, no), aspirin use (no, yes), family history of lung cancer (no, yes, possibly), history of hypertension (no, yes), history of diabetes (no, yes), history of chronic bronchitis (no, yes), history of emphysema (no, yes).

### 3.3 Association between LC mortality and MQI

**Figure 4:**
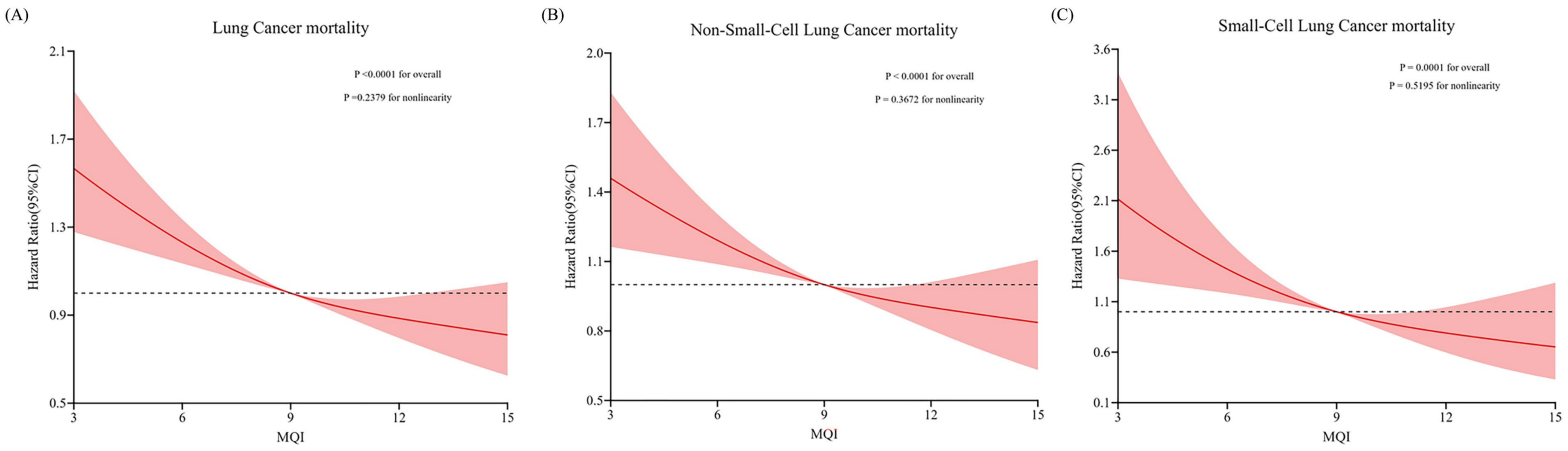
Restricted cubic spline (RCS) model on the association of MQI with mortality of LC **(A)** NSCLC **(B)** and SCLC **(C)**. Hazard ratio was adjusted for age (years), sex (male, female), race (white and non-white), education levels (college below, college graduate, postgraduate), marital status (married, unmarried), smoking status (never, currently/ever), number of cigarettes smoked (0, 1–20, >20 cigarettes/day), history of alcohol consumption (yes, no), history of emphysema (yes, no), chronic bronchitis history (yes, no), body mass index (kg/m^2^), trial arm (intervention, control), aspirin use (yes, no), history of diabetes (yes, no), history of hypertension (yes, no) and family history of lung cancer (yes, no).

During an average 15.07 ± 4.54-year follow-up (1,533,359 person-years), 1,217 deaths attributed to LC were identified, including 1,005 cases of NSCLC and 212 cases of SCLC, with an overall mortality rate of about 7.94 per 10,000 person-years. As shown in Table 4, after adjusting for potential confounders, Cox regression analysis revealed a significant inverse link between higher MQI and LC mortality (HR Q4 vs. Q1: 0.71; 95% CI: 0.60, 0.84. *p* < 0.001 for trend). Similar negative correlations were observed in the association between MQI and the mortality of NSCLC (HR Q4 vs. Q1: 0.75; 95% CI: 0.62, 0.90; *p* = 0.001 for trend) and SCLC mortality (HR Q4 vs. Q1: 0.56; 95% CI: 0.36, 0.85; *p* = 0.001 for trend). The RCS model revealed linear relationships between MQI and the mortality of overall LC and both NSCLC and SCLC (Figure 4). The survival curve results reveal significant differences in survival curves among different MQI level groups (*p* < 0.0001). As time progresses, the high-MQI group exhibits a generally lower mortality rate of lung cancer overall (Supplementary Figure 2).

**Table 4:** Hazard ratios of the association between MQI and lung cancer mortality.

Quartiles of MQI	Cases	Person-years	Incidence rate per 10,000 person-years (95% confidence interval)	Hazard ratio (95% confidence interval) by MQI		
				Unadjusted	Model 1^a^	Model 2^b^
Lung cancer						
Quartile 1	541	531975.3	10.17(9.35,11.06)	1.000 (reference)	1.000 (reference)	1.000 (reference)
Quartile 2	290	358332.2	8.09(7.21,9.08)	0.80 (0.70, 0.93)	0.86 (0.74, 0.99)	0.90 (0.78, 1.03)
Quartile 3	204	323206.2	6.31(5.50,7.24)	0.63 (0.54, 0.74)	0.69 (0.59, 0.82)	0.75 (0.63, 0.88)
Quartile 4	182	319845.3	5.69(4.92,6.58)	0.57 (0.48, 0.67)	0.65 (0.55, 0.77)	0.71 (0.60, 0.84)
*p* for trend				<0.001	<0.001	<0.001
Non-small-cell lung cancer						
Quartile 1	429	531975.3	8.06 (7.34, 8.86)	1.000 (reference)	1.000 (reference)	1.000 (reference)
Quartile 2	250	358332.2	6.98 (6.16, 7.90)	0.87 (0.75, 1.02)	0.93 (0.79, 1.09)	0.97 (0.83, 1.14)
Quartile 3	172	323206.2	5.32 (4.58, 6.18)	0.67 (0.56, 0.80)	0.74 (0.62, 0.88)	0.79 (0.66, 0.95)
Quartile 4	154	319845.3	4.82 (4.11, 5.64)	0.61 (0.51, 0.73)	0.69 (0.57, 0.83)	0.75 (0.62, 0.90)
*p* for trend				<0.001	<0.001	0.001
Small-cell lung cancer						
Quartile 1	112	531975.3	2.11 (1.75, 2.53)	1.000 (reference)	1.000 (reference)	1.000 (reference)
Quartile 2	40	358332.2	1.12 (0.82, 1.52)	0.54 (0.37, 0.77)	0.57 (0.40, 0.82)	0.60 (0.42, 0.86)
Quartile 3	32	323206.2	0.99 (0.70, 1.40)	0.48 (0.32, 0.71)	0.53 (0.36, 0.79)	0.58 (0.39, 0.86)
Quartile 4	28	319845.3	0.88 (0.61, 1.27)	0.43 (0.28, 0.65)	0.49 (0.32, 0.75)	0.56 (0.36, 0.85)
*p* for trend				<0.001	<0.001	0.001

^a^Model 1 was adjusted with age (continuous), sex (male, female), race (white, non-white), education levels (college below, college graduate, postgraduate) and marriage (married, unmarried).

^b^Model 2 was adjusted for model 1 plus BMI at baseline (continuous), trial arm (intervention, control), smoking status (never, current or former), daily cigarette consumption (0, 1–20, >20), history of alcohol consumption (yes, no), aspirin use (no, yes), family history of lung cancer (no, yes, possibly), history of hypertension (no, yes), history of diabetes (no, yes), history of chronic bronchitis (no, yes) and history of emphysema (no, yes).

Subgroup analysis detected a significant interaction (*p* < 0.05) regarding the influence of BMI on the inverse relationship between MQI and LC mortality. This implies that a higher MQI confers a more pronounced protective effect in mitigating LC mortality among participants BMI ≤ 30 (HR Q4 vs. Q1: 0.67; 95% CI: 0.56, 0.81; *p* < 0.001 for trend) compared to participants BMI > 30 (HR Q4 vs. Q1: 1.01; 95% CI: 0.68, 1.51; *p* = 0.8769 for trend). In other subgroup analyses, no substantial differences in the inverse relationship between MQI and LC incidence were found (all interaction *p*-values > 0.05) (all interaction *p*-values > 0.05), as detailed in Supplementary Table 3. Sensitivity analyses demonstrated a significant robust negative association between MQI value and LC mortality (Table 5).

**Table 5:** The sensitivity analyses between MQI and lung cancer mortality.

Categories	HR^e^ (Quartile 4 vs. Quartile 1, 95% CI)	*p* for trend
Exclude extreme energy intake^a^	0.73 (0.61,0.88)	<0.001
Exclude extreme BMI^b^	0.71 (0.60,0.85)	<0.001
Excluding Patients diagnosed within 2 years	0.69 (0.57,0.83)	<0.001
Excluding Patients diagnosed within 4 years	0.67 (0.54,0.82)	<0.001
Excluding patients with diabetes or respiratory comorbidities^c^	0.69 (0.57,0.84)	<0.001
Replace the Indicator of cigarettes smoked^d^	0.79 (0.67,0.94)	0.002

^a^Extreme energy intake was defined as energy intake >4,000 kcal/day or <500 kcal/day.

^b^BMI defined as body mass index at baseline (kg/m^2^).

^c^Respiratory comorbidities included contain emphysema and chronic bronchitis.

^d^Adjusting for pack-years of smoking (continuous) instead of daily cigarette consumption (0, 1–20, or >20).

^e^HR was adjusted for age (years), sex (male, female), race (white and non-white), education levels (college below, college graduate, postgraduate), marital status (married, unmarried), BMI at baseline (continuous), trial arm (intervention, control), smoking status (never, current or former), daily cigarette consumption (0, 1–20, >20), history of alcohol consumption (yes, no), aspirin use (no, yes), family history of lung cancer (no, yes, possibly), history of hypertension (no, yes), history of diabetes (no, yes), history of chronic bronchitis (no, yes), history of emphysema (no, yes).

## 4 Discussion

Drawing on data from the PLCO cancer screening trial, this research investigates the associations between MQI values and the incidence and mortality of LC. Our findings indicate that elevated MQI values correlate with a reduced incidence of LC, encompassing NSCLC and SCLC. Furthermore, a comparable inverse relationship was observed between MQI and the risk of LC mortality. These correlations remained statistically significant even after adjusting for a range of potential confounders, such as lifestyle factors and demographic variables. The RCS model demonstrated linear inverse relationships for both the incidence and mortality of overall LC, as well as its NSCLC and SCLC subtypes. Sensitivity analyses affirmed the robustness of our results.

Our subgroup analyses revealed significant interactions (both *p* < 0.05) in how smoking status and BMI modify the inverse relationships between the MQI and LC outcomes. For smoking status, the MQI’s protective effect against LC incidence was markedly stronger in current or former smokers. This could be due to the fact that smokers are exposed to a high load of carcinogens, and a diet with a higher MQI, likely rich in antioxidants, anti-inflammatory agents, and nutrients that support DNA repair and immune function, may help counteract the oxidative stress, inflammation, and DNA damage induced by smoking. These beneficial substances may enhance the body’s ability to clear carcinogens, repair damaged cells, and mount an effective immune response against pre-cancerous or cancerous cells, thus offering greater protection in this high-risk group (40, 41). Moreover, regarding BMI, a more pronounced protective effect of a higher MQI on LC mortality was observed in participants with BMI ≤ 30. Individuals with a normal BMI often have a more efficient metabolic profile, allowing for better absorption and utilization of the nutrients in a high-MQI diet. In contrast, obesity is associated with metabolic dysregulation, chronic low-grade inflammation, and altered immune function, which may disrupt the normal physiological pathways through which MQI-related factors exert their protective effects, thereby diminishing the MQI-LC mortality association in the high-BMI group (42–44).

The inverse correlation between MQI and LC risk underscores a multi-faceted dietary strategy that leverages antioxidant-rich plant foods, anti-inflammatory fats, and metabolic regulators—among other elements—to collectively disrupt carcinogenesis. High-MQI diets prioritize nutrient synergy over isolated components: their abundance of polyphenols (from fruits, herbs, olive oil), dietary fiber (from whole grains, legumes), and unsaturated fats (from nuts, fish) forms a systemic defense network that operates through multiple interconnected layers (10, 21, 43). First, oxidative stress is mitigated via polyphenol-driven free radical scavenging and fiber-mediated gut microbiome modulation. Polyphenols neutralize reactive oxygen species (ROS) generated by environmental carcinogens (e.g., tobacco smoke) (41, 45), while fermentation of dietary fiber in the colon produces short-chain fatty acids (SCFAs) that strengthen intestinal barriers, thereby preventing microbial toxins from triggering lung inflammation (46). Second, chronic inflammation is suppressed through fat quality optimization and insulin regulation. High-MQI diets replace saturated fats (e.g., red meat, butter) with omega-3 fatty acids (e.g., salmon, walnuts) and monounsaturated fats (e.g., olive oil), reducing pro-inflammatory cytokine production (e.g., TNF-α, IL-6) and enhancing insulin sensitivity (47, 48). Lower insulin levels, in turn, disrupt cancer cell proliferation by inhibiting growth signaling pathways (49). Third, metabolic adaptation is promoted via carbohydrate restriction and nutrient density. High-MQI diets’ emphasis on low-glycemic foods (e.g., legumes, non-starchy vegetables) stabilizes blood glucose, limiting insulin surges that fuel tumor metabolism, while their nutrient-dense profile (high vitamins/minerals, low refined sugars) creates a hostile metabolic environment for LC cells by restricting access to energy substrates (e.g., glucose, methionine) and enhancing immune surveillance (13, 50). This integrated approach also exploits inter-organ communication: SCFAs from gut fermentation enter circulation to directly suppress lung inflammation, while polyphenols absorbed in the small intestine reach the lungs to inhibit angiogenesis—a critical process for tumor expansion (51). However, the protective efficacy of high-MQI diets is context-dependent: individuals with pre-existing lung damage (e.g., smokers) may require complementary interventions (e.g., smoking cessation) to fully benefit, as dietary effects alone cannot reverse chronic carcinogen exposure (40). Ultimately, high-MQI diets reduce LC risk by transforming the body’s internal terrain from a tumor-permissive to a tumor-resistant state through sustained dietary patterning, rather than relying on transient nutrient spikes.

In the field of LC prevention and treatment, the high MQI diet provides crucial directions for the refinement of practice guidelines and the optimization of patient management. Regarding practice guidelines, protein intake should emphasize the high quality of sources. Priority should be given to foods such as lean meat, fish, and legumes, which are rich in essential amino acids and have high bioavailability. This helps meet the body’s requirements for normal metabolism and the maintenance of immune function, thereby reducing adverse metabolic states associated with an increased risk of lung cancer. For fat intake, attention should be paid to the rational proportion of fatty acids. It is advisable to increase the proportion of monounsaturated fatty acids and polyunsaturated fatty acids while reducing the intake of saturated fatty acids and trans-fatty acids. This approach can regulate the body’s inflammatory response, improve cell membrane function, and create a favorable internal environment for lung health. As for carbohydrates, preference should be given to whole grains and tubers that are high in dietary fiber and have a low glycemic index. These foods help stabilize blood sugar levels, control body weight, and avoid pro-carcinogenic factors triggered by blood sugar fluctuations and obesity. In terms of patient management, personalized dietary plans should be formulated based on the aforementioned MQI diet principles according to the different treatment stages of lung cancer patients. Before surgery, precise adjustments to protein, fat, and carbohydrate intake can enhance the patient’s physical reserves and immune function, thereby improving surgical tolerance. During the post-operative period and radiochemotherapy, a reasonable combination of nutrients can alleviate adverse treatment reactions and promote physical recovery and tissue repair. For patients in the advanced stage, a scientific intake ratio can help maintain nutritional balance, improve the quality of life, and prolong survival.

This research boasts several notable strengths that enhance the credibility of its findings. First, the data were derived from a large, prospective cohort of over 100,000 participants with diverse occupational backgrounds, recruited from 10 screening centers across the United States. This expansive and heterogeneous sample ensures broad representativeness, while the study’s extended follow-up period bolsters the reliability of the results. The prospective design of the PLCO study, combined with sensitivity analyses, effectively minimizes the risk of reverse causation, which could occur if subclinical conditions influenced dietary habits. This approach strengthens the validity of the observed associations between dietary factors and LC outcomes. The study also rigorously addressed selection bias by ensuring comparable rates of LC diagnoses between excluded and included participants, thereby enhancing internal validity. After adjusting for multiple potential confounders, the robustness of the study’s conclusions was further reinforced (23, 52). Previous studies on the relationship between diet and LC have predominantly focused on single nutrients or overall intake levels, which presents a limited perspective. This has led to inconsistent and even contradictory results across different studies, making it difficult to form a unified understanding (8, 12, 14). In contrast, this study innovatively employed the MQI to comprehensively evaluate dietary patterns. The MQI takes into account both the synergistic effects among nutrients and differences in their quality, enabling a more precise reflection of the impact of diet on health. Meanwhile, this study paid particular attention to the histological heterogeneity of LC and analyzed the associations between macronutrient quality and the risks of different LC subtypes. This comprehensive and targeted approach represents a key distinction between this study and previous research, offering a novel and more valuable perspective for understanding the complex relationship between diet and LC.

This study has notable limitations that warrant discussion. First, while mode/median imputation ensured computational simplicity and data completeness, it has three main drawbacks: (1) single-value imputation ignores variable distributions, potentially underestimating variability; (2) systematic bias may arise under MNAR conditions; (3) unlike multiple imputation (MI), it fails to quantify missing data uncertainty, risking standard error underestimation. Consequently, future work should consider MI or machine learning methods (e.g., random forest imputation) to improve robustness. Second, the use of a single baseline nutritional assessment introduces the risk of bias over time, as dietary habits are dynamic (53). This approach may fail to capture the cumulative effects of diet on disease incidence, despite baseline assessments generally reflecting habitual long-term intake patterns based on nutritional science. However, the relatively short DHQ employed may underestimate dietary intake variability (34). Third, like most observational studies, residual confounding from unmeasured factors cannot be entirely excluded, potentially influencing the observed associations. Fourth, self-reported dietary data are prone to recall bias, which may compromise the accuracy of dietary exposure assessments. Fifth, while the study population primarily consisted of middle-aged and elderly Americans, the generalizability of the MQI-LC associations to other populations or age groups remains unclear. Further research is needed to explore these relationships across diverse demographics and identify subgroup-specific differences. Moreover, in the subgroup analyses of this study, we did not employ traditional multiple-testing correction methods such as the Bonferroni correction or False Discovery Rate control, which presents certain limitations. Due to the multiple subgroup tests conducted, the risk of Type I error inflation increases, meaning that false – positive subgroup association results may occur. Although we assessed interaction *P*-values to detect potential false subgroup effects, the lack of correction makes the results more susceptible to random errors. This may lead to the observed associations in some subgroups not being real but rather due to chance factors in multiple testing. Therefore, extreme caution is needed when interpreting the subgroup analysis results, and further research is required to validate these findings in the future. Additionally, this study was subject to participant attrition, and the potential for attrition bias may pose a threat to the internal validity of the research findings. Finally, given the observational design, causal interpretations of the diet-cancer associations must be made cautiously, as residual or unmeasured confounding could influence the findings.

## 5 Conclusion

Given the current body of evidence and the complex interrelationships among diet, physical activity, and body composition, dietary patterns with higher MQI should be regarded as a fundamental component of a holistic, healthy lifestyle, rather than an isolated or modifiable risk factor. Collectively, this research highlights a significant inverse association between elevated MQI and reduced incidence and mortality rates of LC. These findings offer novel insights into dietary approaches for preventing and managing LC, providing a robust scientific foundation for developing evidence-informed, sustainable dietary guidelines and public health interventions.

## Data Availability

The original contributions presented in the study are included in the article/Supplementary material, further inquiries can be directed to the corresponding authors.

## Footnotes

^1^ https://cdas.cancer.gov/learn/plco/early-qx/
